# 562 Therapy Resources for Management of Large TBSA Burns: A Case Study

**DOI:** 10.1093/jbcr/irad045.158

**Published:** 2023-05-15

**Authors:** Audrey O'Neil, Cassandra Rush, David Roggy, Brett Hartman

**Affiliations:** Richard M. Fairbanks Burn Center, Indianapolis, Indiana; Eskenazi Health, Indianapolis, Indiana; Indiana university/Eskenazi Health, Indianapolis, Indiana; Richard M. Fairbanks Burn Center, Indianapolis, Indiana; Eskenazi Health, Indianapolis, Indiana; Indiana university/Eskenazi Health, Indianapolis, Indiana; Richard M. Fairbanks Burn Center, Indianapolis, Indiana; Eskenazi Health, Indianapolis, Indiana; Indiana university/Eskenazi Health, Indianapolis, Indiana; Richard M. Fairbanks Burn Center, Indianapolis, Indiana; Eskenazi Health, Indianapolis, Indiana; Indiana university/Eskenazi Health, Indianapolis, Indiana

## Abstract

**Introduction:**

Therapy studies involving large percent TBSA burns ( >50% TBSA) are not well described. Studies show the highest predictor of preventing burn scar contracture is the amount of burn rehabilitation time received per cutaneous functional units involved, but what does “more therapy time” mean for larger burns in terms of therapy resources for burn centers?

**Methods:**

A case study was completed on a 20-year-old male admitted after sustaining 85% TBSA full thickness flame burns to his face, neck, arms, legs, and trunk, along with an inhalation injury. He was admitted to the inpatient burn ICU for 113 days, undergoing 16 surgeries to achieve skin coverage prior to discharge to acute rehab. Therapy was initiated < 24 hours after admission to the Burn ICU. Direct therapy contact minutes were tracked during all session and divided into therapy treatment domains. (resource). If co-treating occurred, a single unit of time was used for the time of the session to avoid doubling the units provided.

**Results:**

Therapy occurred on 106/113 days of admission, receiving 238 therapy sessions (113 PT, 125 OT). He received 21,027 minutes of direct therapy, averaging 196.5 min/rehab days. The highest number of therapy minutes were spent on ROM (9,032 min). Other domains of care included Mobilization (3,171 min), Positioning (2,298 min), Orthotic management (1,326 min), etc. (Table 1). Despite focus on ROM and positioning, he discharged with 7 moderate (left wrist extension, bilateral hip abduction, bilateral ankle dorsiflexion, left neck rotation, and left forearm supination) and 4 severe contractures (right wrist extension, bilateral hip extension, neck extension). He was able to transfer with moderate assistance and walk 150 ft with a platform walker at discharge.

**Conclusions:**

Therapy interventions in the literature are often described in isolated “domains” of practice, however guidelines on a global approach to management of those with catastrophic injuries does not exist. Prioritization between domains with limited therapy resources when facing patients with extensive deficits is challenging. A dedicated burn therapy staff with availability to secure additional resources was required to ensure optimal outcomes in the management of this patient

**Applicability of Research to Practice:**

This case study demonstrates the comprehensive burden of care and need for additional therapy resources in the management large TBSA burns.

